# An Unexpected Iron (II)-Based Homogeneous Catalytic System for Highly Efficient CO_2_-to-CO Conversion under Visible-Light Irradiation

**DOI:** 10.3390/molecules24101878

**Published:** 2019-05-16

**Authors:** Zi-Cheng Fu, Cheng Mi, Yan Sun, Zhi Yang, Quan-Qing Xu, Wen-Fu Fu

**Affiliations:** 1 College of Chemistry and Engineering, Yunnan Normal University, Kunming 650092, China; zichengfu101@sina.cn (Z.-C.F.); micheng121@sina.com (C.M.); sunyan19931110@163.com (Y.S.); kmyangz@sina.com (Z.Y.); qqxu1977@163.com (Q.-Q.X.); 2 Key Laboratory of Photochemical Conversion and Optoelectronic Materials and HKU-CAS Joint Laboratory on New Materials, Technical Institute of Physics and Chemistry, Chinese Academy of Sciences, Beijing 100190, China

**Keywords:** Fe-based catalyst, Ru(II) complex, photocatalysis, CO_2_ reduction, CO

## Abstract

We present two as-synthesized Fe(II)-based molecular catalysts with 1,10-phenanthroline (phen) ligands; Fe(phen)_3_Cl_2_ (**1**) and [Fe(phen)_2_(CH_3_CH_2_OH)Cl]Cl (**2**), and their robust catalytic properties for the conversion of CO_2_ to CO in DMF/TEOA (DMF = *N*,*N*’-dimethylformamide; TEOA = triethanolamine) solution containing Ru(bpy)_3_^2+^ and BIH (1,3-dimethyl-2-phenyl-2,3- dihydro-1*H*-benzo-[d]-imidazole). High turnover numbers (TONs) of 19,376 were achieved with turnover frequencies (TOFs) of 3.07 s^−1^ for complex **1** (1.5 × 10^−7^ M). A quantum efficiency of 0.38% was observed after 5 h irradiated by 450 nm monochromatic light. The generation rate of CO_2_ and H_2_ were tuned by optimizing the experimental conditions, resulting in a high CO selectivity of 90%. The remarkable contribution of the photosensitizer to the total TON_CO_ was found being 19.2% (as shown by tests under similar conditions without catalysts) when BIH was employed as a sacrificial electron donor. The product selectivity in complex **2** reached 95%, and the corresponding TON_CO_ and TOF_CO_ were 33,167 and 4.61 s^−1^ in the same concentration with complex **1** used as catalyst; respectively. This work provides guidance for future designs of simple, highly efficient and selective molecular catalytic systems that facilitate carbon-neutral solar-to-fuel conversion processes

## 1. Introduction

Solar-light-driven reduction of CO_2_ into energy-rich carbon-based products has attracted wide attention, with specific importance attached to the development of highly efficient catalysts and the establishing new photocataytic systems [1,2]. However, activation and conversion of CO_2_ into desirable fuel or feedstock chemicals driven by visible light remains a significant challenge [3,4,5,6,7]. One way to promote the transformations is to employ heterogeneous [8,9,10] or homogeneous catalyst [11,12,13]. In 1986, Lehn and co-workers reported the photocatalytic reduction of CO_2_ with Co polypyridine complex as the catalyst in a homogeneous system [14]. Whereafter, a series of Ru(II)-Re(I) binuclear complexes linked by bridging ligands were then studied by Ishitani’s group, and the TON of photocatalytic reduction of CO_2_ to CO was increased from previously reported 170 to 232 [15,16]. Nevertheless, it is economic and popular to utilize the noble-metal-free complexes as catalysts rather than noble metal. In the regard, homogeneous catalytic systems with excellent potential in visible-light-driven reduction of CO_2_ to CO [17,18,19] were developed using Fe [9,20,21,22], Co [23,24,25], Ni [26,27,28], Cu [29,30,31] and Mn [32,33,34] complexes as catalysts.

A stunning homogeneous molecular system containing nickel *N*-heterocyclic carbene-isoquinoline complex employed as catalyst, iridium complex as photosensitizer (PS) towards photocatalytic transformation of CO_2_ to CO was successfully established [35]. This system exhibited a high photocatalytic performance of CO_2_ to CO with TONs up to 98,000 and a TOF reaching 3.9 s^−1^ for a very low concentration of the catalyst, implying high catalytic sensitivity to the CO_2_ reduction reaction. Recently, Lau and co-workers reported another highly efficient and selective system that consists of Ru(bpy)_3_^2+^, Fe(II) or Co(II) quaterpyridine complex in the presence of two electron donors; BIH, the higher TON_CO_ than 3000 with up to 95% selectivity was observed for the iron catalyst [36]. Due to rare characteristics of Ru and Ir, the homogeneous system involving earth-abundant metal complexes for photocatalytic reduction of CO_2_ was then constructed by Ishitani’s group using Cu(I) Cu(dmp)(P)_2_^+^ (dmp = 2,9-dimethyl-1,10-phenanthroline; P = phosphine ligand) as the PS and [Fe(dmp)_2_(NCS)_2_]^2+^ as the catalyst [37]. The TON_CO_ of 273 and quantum yield of 6.7% were observed with selectivity of the CO up to 70.5%. Among molecular catalysts for the CO_2_-to-CO conversion, a macrocyclic amine cryptate dinuclear cobalt complex has been shown to be robust, using Ru(phen)_3_^2+^ as the PS, the TON reached 16,896 with a quantum efficiency of 0.04% [38].

**Scheme 1:**
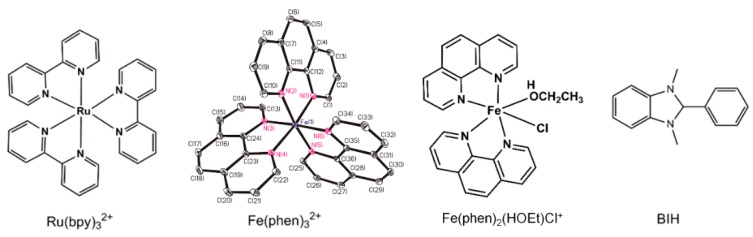
Structures of the photosensitizer, catalysts, and BIH.

Herein, we report a highly efficient and selective homogeneous visible-light-driven catalytic system for CO_2_-to-CO conversion. This includes as-synthesized Fe-based complexes employed as the catalysts, Ru(II) complex as the PS and BIH as the electron donor in DMF/TEOA (Scheme 1). An investigation into catalytic efficiency and selectivity between CO and H_2_ was performed for various concentrations of catalyst, PS and BIH, and different ratios of DMF/TEOA. The corresponding mechanism was also suggested.

## 2. Results and Discussion

### 2.1. Characterization of Catalysts and Electrochemical Property

Fe(phen)_3_Cl_2_ (**1**), [Fe(phen)_2_(CH_3_CH_2_OH)Cl]Cl (**2**) and BIH [39] were prepared according to a modification of published procedures. All of these compounds were characterized by MS, ^1^H NMR spectroscopy and elemental analysis (see the section of materials and methods, Figure S1 in the Supplementary Materials). The crystal structure of Fe(phen)_3_Cl_2_ was determined by single-crystal X-ray diffraction analysis (Figure S2 and Table S1). The cyclic voltagrammetry of Fe(phen)_3_Cl_2_ was recorded in a DMF solution containing 0.1 M ^n^Bu_4_NPF_6_ under an Ar atmosphere at ambient temperature (Figure S3). Figure S3 shows three distinct redox waves at −1.05, −1.21 and −1.44 V vs. NHE, with these tentatively attributed to Fe^II^(phen)_3_/Fe^I^(phen)_3_, Fe^I^(phen)_3_/Fe^0^(phen)_3_ and Fe^0^(phen)_3_/Fe^0^(phen)_2_(phen^∙−^), respectively [40]. After CO_2_ bubbling to the system, two irreversible reduction waves underwent slight negatively shifts from −1.05 and −1.21 V to −1.08 and −1.32 V, respectively. There was a remarkable current enhancement for the latter, consistent with the electrocatalytic CO_2_ reduction (Figure S3) [41,42].

### 2.2. Catalyst-Concentration Dependence of Phtocatalytic CO_2_-to-CO Conversion for Fe(phen)_3_Cl_2_ (1) 

**Figure 1:**
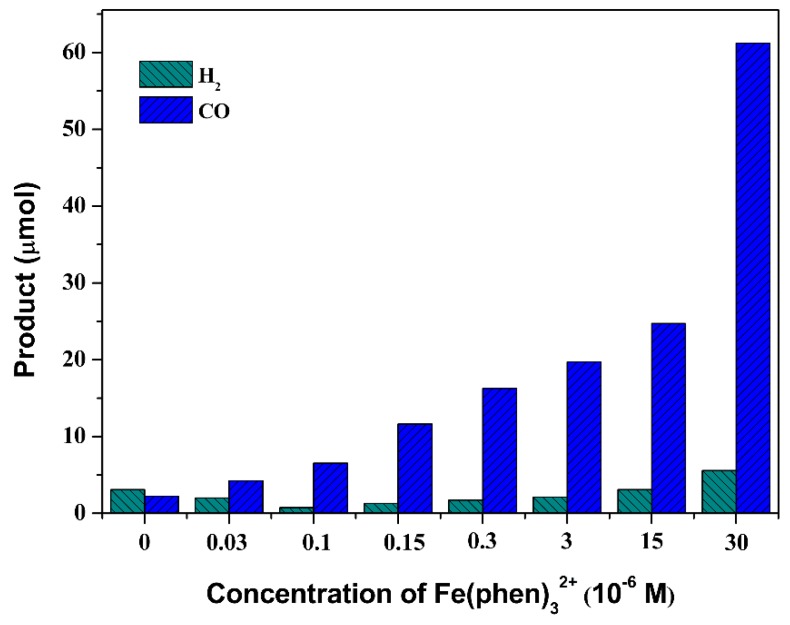
Catalyst-concentration dependence of CO and H_2_ production in 4 mL CO_2_-saturated DMF/TEOA solution (*v*/*v*, 7:1) containing 6.7 × 10^−4^ M Ru(bpy)_3_^2+^ and 0.022 M BIH after bright LED visible-light irradiation for 2 h at 298 K.

The photocatalytic reduction systems were assembled using Ru(bpy)_3_^2+^, complex **1** and BIH in a CO_2_-saturated DMF/TEOA solution. The produced gas products were identified using gas chromatography (GC), with Ar as carrier gas. The generating rates of CO and H_2_ were determined as a function of catalyst concentration. The results in Figure 1 clearly show that the production of CO and H_2_ was enhanced with increasing catalyst concentrations, and reaction system was accompanied by a H_2_-generating reaction that becomes less important at higher catalyst concentrations. In comparison to results for a lower catalyst concentration (3.0 × 10^−8^ M) where 4.25 µmol CO was produced with a selectivity of 68% after 2 h of visible-light irradiation, and a higher catalyst concentration of 3.0 × 10^−5^ M evolved 61.21 µmol CO at a selectivity of 92% (Table 1). Furthermore, 96.23 µmol of CO and a selectivity of 94% were achieved when the concentration of Fe(phen)_3_Cl_2_ reached 1.5 × 10^−4^ M, which clearly show that concentration of catalyst plays significant role to selectivity of CO_2_-to-CO conversion. However, in the absence of the Fe(II) complex, small amounts of CO and H_2_ were detected, with the H_2_-generation rate being superior to that of CO_2_ reduction [43,44,45], indicating a significant contribution by the PS to the CO-generating rate and the effect on product selectivity (Table 1, entry 4) [46].

### 2.3. The Dependence of the Photocatalytic Reduction Rates on PS for Catalyst (1)

To evaluate the catalytic capacity of the catalysts towards CO_2_ reduction and accompanied H_2_ production, the dependence of the photocatalytic reduction rates on PS concentration was conducted at a fixed concentration of Fe(phen)_3_Cl_2_ and various irradiation time. Figure S4 demonstrates that higher conversion efficiencies of CO_2_-to-CO were achieved after 6 h irradiation of the reaction system containing Fe(phen)_3_Cl_2_ (1.5 × 10^−7^ M) and BIH (0.022 M) with various PS concentrations. Increasing the amount of PS from 1.7 × 10^−4^ to 6.7 × 10^−4^ M significantly enhanced CO_2_ conversion and H_2_ evolution (Figure S5), with produced amounts of CO and H_2_ increasing from 1.64 and 1.74 µmol to 16.63 and 3.45 µmol, respectively (Figures S4 and S5). However, the CO selectivity of 83% (irradiation time for 6 h), when compared with that for a solution at the same concentration of PS and catalyst (Table 1, entry 2), clearly showed that the main-product selectivity decreased for longer irradiation times, suggesting a change of catalytic active species during photochemical reaction. This probably results from the contribution of generated Ru^I^(bpy)_3_^+^ species during photocatalytic reaction to CO_2_-to-CO conversion and H_2_ evolution. 

### 2.4. Optimization of Reaction Conditions for Photocatalysis

Furthermore, optimized conditions for product selectivity were considered in a CO_2_-saturated DMF/TEOA solution (*v/v*, 2.5:1) containing fixed amounts of PS and Fe(phen)_3_Cl_2_ at various BIH concentrations. As shown in Figure S6, the amount and selectivity of CO production strongly depends on the concentration of BIH and enhanced with increasing BIH concentration, reaching a limiting value at a scope of 0.022 M of BIH. Subsequent addition of BIH led to a negative relationship of photocatalytic efficiency, and H_2_ even become dominant. Previous studies revealed that metal nanoparticles such as Fe [47] and Ni [48] may possibly be involved in the homogenous photocatalytic reduction reaction. Thus, considering that BIH has a stronger reducing ability (*E*^ox^_1/2_ = 0.33 V vs. SCE), the possibility of in situ formation of Fe or Ru nanoparticles was assessed by a dynamic light scattering experiment [39,49]. The analysis confirmed that nanosized particles were not found during the photocatalytic reaction (Figure S7). The influence of BIH concentration on the photocatalytic efficiency and product selectivity possibly originates from its intense reduction and proton transfer ability, changing the relative proportion of some active components in the system during the photochemical reaction. Additionally, we tentatively contributed the changes to involving effective one-electron transfer from BIH to the excited state of PS and subsequent electron transfer from BI^•^ to the related species such as Ru(II)*, Ru(I) or Fe(II) catalyst with proton loss from BIH^•+^ (see mechanistic interpretation). Compared with catalytic activity of Fe-based complex for CO_2_-to-CO conversion, the accumulation of generated Ru(I) species as catalyst in the case of high concentration of BIH is disadvantageous for reduction efficiency of CO_2_ in the investigated system [49]; a detailed study is still in progress. Further controlled experiments showed that no CO and H_2_ were generated when any of the following components were absent: light, CO_2_ or TEOA (Table 1).

**Figure 2:**
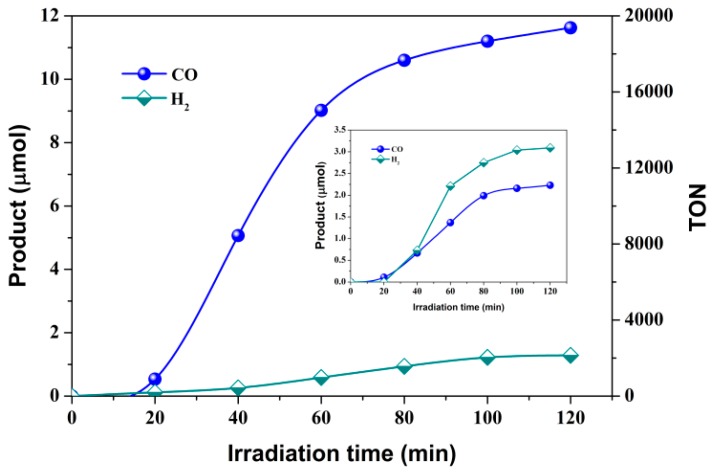
Evolution of CO (blue) and H_2_ (dark cyan), and TON_CO_ during bright LED visible-light irradiation in 4 mL CO_2_-saturated DMF/TEOA solution (*v*/*v*, 7:1) containing 6.7 × 10^−4^ M Ru(bpy)_3_^2+^, 0.022 M BIH and 1.5 × 10^−7^ M Fe(phen)_3_^2+^. Inset: without Fe(phen)_3_^2+^.

As discussed above (Figure S6), the optimized concentration of BIH was 0.022 M. A typical example of the products as a function of irradiation time for a CO_2_-saturated solution with and without catalyst was given in Figure 2. Upon bright white LED irradiation, little CO and H_2_ were produced in the initial 15 min. We assumed that during this period, the phen ligand attached to the central Fe underwent photoinduced partial dissociation and/or a TEOA-substitution reaction to generate active-site-bearing catalytic species [50]. Figure 2 clearly demonstrates changes in the relative amounts of products for the competing pathways with time. Following the initial period, a higher CO-generating rate was observed. A TON of 19,367 was achieved under subsequent 105 min of light irradiation, with a TOF of 3.07 s^−1^ and a CO selectivity that reached 90% (Table 1, entry 2). Under the same conditions, the quantum yield of the photocatalytic reduction of CO_2_ to CO was measured as 0.38% after 5 h of irradiation with 450 nm monochromatic light (Xe lamp, 15 A). The inset in Figure 2 demonstrates that although the contribution of PS to the total catalytic efficiency was less important (2.23 µmol of CO, 3.09 µmol of H_2_), in the parallel catalytic reduction processes without Fe(phen)_3_Cl_2_ the Ru(II) complex exhibited a relatively high catalytic activity for H_2_ generation. The amount of H_2_ generated for the catalytic system compared with those in the system containing catalyst suggested that Fe(phen)_3_Cl_2_ exhibited higher selectivity to CO than to H_2_. Besides, the dependence of the calculated TON_CO_ on the catalyst concentration was not linear because of the sluggish kinetics involving multiple electrons and protons. High TONs of up to 35,417 after 2 h of irradiation were observed for Fe(phen)_3_Cl_2_ concentration as low as 3.0 × 10^−8^ M, which is consistent with previous results [35,36,51].

### 2.5. Comparison of Catalytic Efficiencies of CO_2_-to-CO Conversion for Catalysts 1 and 2 and Isotopic Labeling Experiment

**Figure 3:**
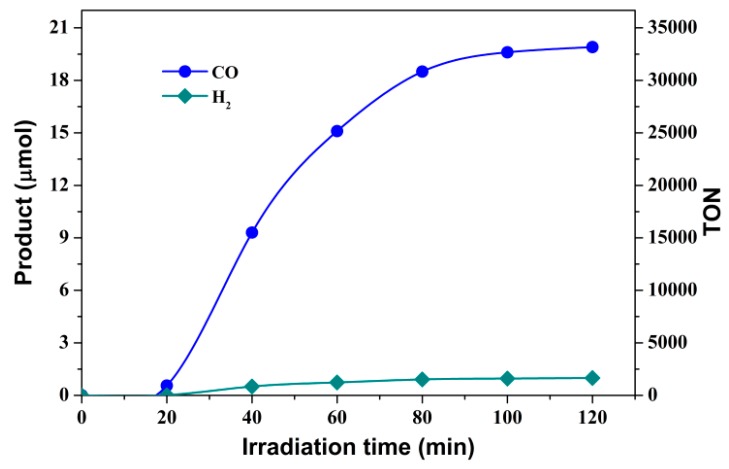
Visible-light-driven production of CO (blue) and H_2_ (dark cyan) in 4 mL CO_2_-saturated DMF/TEOA solution (*v/v*, 7:1) containing 6.7 × 10^−4^ M Ru(bpy)_3_^2+^, 0.022 M BIH and 1.5 × 10^−7^ M Fe(phen)_2_(CH_3_CH_2_OH)Cl^+^.

The catalytic activity of [Fe(phen)_2_(CH_3_CH_2_OH)Cl]Cl (**2**) was then evaluated under the same conditions. A comparison of the CO-evolution over 2 h of irradiation demonstrated that catalytic activity of complex **2** for the transformation of CO_2_ to CO was greater than that of complex **1** (Figure 3). An unexpected high TON of 33,167 was achieved with a TOF of 4.61 s^−1^ for 1.5 × 10^−7^ M of catalyst **2**. When the catalyst concentration was increased to 3.0 × 10^−5^ M, 119.86 µmol of CO was generated, almost twice the number of times than that produced by catalyst **1** (61.21 µmol), with a CO selectivity of over 85% (Table S2). A comparison of TON_CO_ for catalytic performance of CO_2_ reduction between catalyst **2** and Fe(II) quaterpyridine complex of previous report in different solvents [36], TON_CO_ values of 9,988 for the former (3.0 × 10^−5^ M, Table S2) and 1,879 for the latter (5.0 × 10^−5^ M) in the presence of Ru(bpy)_3_^2+^ and BIH, demonstrates that catalyst **2** used in the work exhibits higher catalytic activity for CO_2_ reduction. Additionally, we found that the contribution of PS to CO_2_-to-CO conversion is remarkable in the absence of Fe(II) complex catalyst when BIH is used as an electron donor, and the effect is enhanced with increasing concentration of BIH and the change of DMF/TEOA ratio (inset in Figure 2 and Figure S8). In order to identify the origin of the produced CO, an isotopic labeling experiment was conducted using ^13^CO_2_ as the substrate on a GC-MS system under identical catalysis conditions [52,53]. As shown in Figure S9, the peak with m/z = 29 could be assigned to the reduced product of ^13^CO_2_ and the m/z value of 45 was the original ^13^CO_2_. These results confirmed that the CO evolution originated from the photocatalytic reduction of CO_2_ and precluded possible degradation of organics used.

### 2.6. Detection of Reaction Intermediates and Mechanistic Interpretation

**Figure 4:**
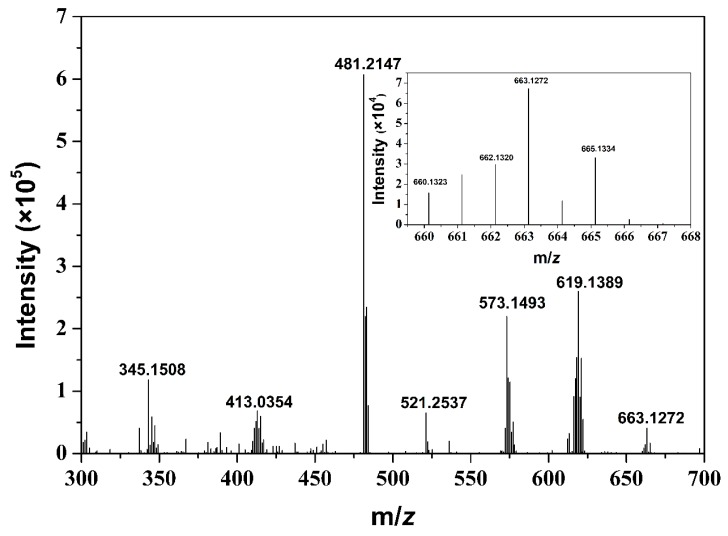
HRMS spectra of CO_2_-saturated DMF/TEOA solution (*v*/*v*, 7:1) containing 0.67 mM Ru(bpy)_3_^2+^, 0.022 M BIH and 1.5 × 10^−7^ M Fe(phen)_3_^2+^ after irradiation for 30 min. Inset: measured isotopic distribution.

**Scheme 2:**
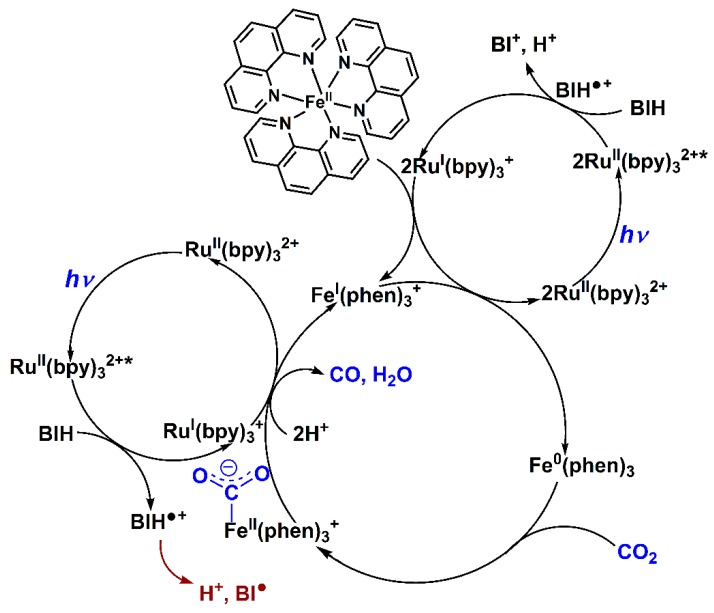
Proposed mechanism of the photocatalytic CO_2_-to-CO conversion for the presented system containing catalyst (**1**), [Ru(bpy)_3_]^2+^ and BIH.

The detection and characterization of stable intermediates during the photoinduced reduction of CO_2_ are of fundamental importance to the identification of the reaction mechanism leading to CO formation. High resolution mass spectrometry (HRMS) was therefore used to probe active intermediates under the given catalytic conditions. Figure 4 clearly shows a signal at *m*/*z* = 663.1272 in the positive mode, which was attributed to the intermediate [Fe(phen)_3_ + CO_2_ + Na]^+^. Nevertheless, we did not observe a signal corresponding to [Fe(phen)_2_(TEOA) + CO_2_ + Na]^+^ (*m*/*z* = 632.17). The HRMS results obtained can be considered as clear evidence for the interchange mechanism during CO_2_ photoreduction. On the basis of these results, mechanism for photocatalytic CO_2_-to-CO conversion by Fe-based complexes is proposed in Scheme 2. Upon excitation at visible light, the triplet excited state of PS was reductively quenched by BIH to give the one-electron reduced species, then the Fe(II) catalyst was doubly reduced to the active Fe(0) species. In such a case of lower oxidation state, we cannot distinguish whether the dissociation of ligand from the complex is anterior to formation of Fe-CO_2_ adduct, but the intermediate Fe(phen)_3_∙∙∙CO_2_ is observed by HRMS analysis. Thus, we speculate that this is accompanied by the contribution of one Fe-N bond breaking and Fe-CO_2_ bond making in terms of an interchange of the ligand and CO_2_, after which photodriven one-electron transfer from Ru^I^(bpy)_3_^+^ to the adduct takes place, which needs to be protonated to cleave the C–OH bond, give CO, and regenerate Fe^I^(phen)_3_^+^. It is noteworthy that in subsequent catalytic reactions, BI^•^ produced as an intermediate of sacrificial electron donor has strong reducing power (*E*^ox^ = −2.06 V vs. Fc^+^/Fc), and donate performance. This is good agreement with experimental observation that in the initial stage there is remarkable induction period for H_2_ and CO production. This means that the generated BI^•^ not only accelerates the formation of Ru^I^(bpy)_3_^+^ but also probably directly transfers electron to Fe(II) catalyst to produce active catalytic species. Further studies on the contributions of PS to H_2_ and CO evolution are in progress in our laboratory and will bring further insight into the solution of the problem.

## 3. Materials and Methods

### 3.1. Materials

Iron(II) chloride tetrhydrate (98%, Energy Chemical Ltd., Shanghai, China), 1,10-Phenanthroline (99%, J&K Scientific Ltd., Beijing, China), [Ru(bpy)_3_]Cl_2_·6H_2_O, 2-Phenylbenzimidazole (98%, J&K Scientific Ltd., Beijing, China), Iodomethane (97%, Ouhechem, Beijing, China), Sodium borohydride (99%, HuaDa, Guangdong, China). Sodium chloride, Sodium thiosulfate, Ethanol, Dichloromethane, Dithyl ether, *N*,*N*-Dimethylformamide, Triethanolamine come from Beijing Chemical Works (Beijing, China), and were used as purchased, without further purification.

### 3.2. Synthesis

The synthesis of Fe(phen)_3_Cl_2_ (**1**). Catalyst **1** was carried out as follows: FeCl_2_∙4H_2_O (1.00 g, 5.03 mmol) and phen (2.72 g, 15.09 mmol) were added to 300 mL of ethanol and stirred under Ar overnight. The solvent was then removed by evaporation under vacuum. The solid product was purified by recrystallization at least four times in ethanol and ether. Yield: 90% (3.02 g). ^1^H NMR (400 MHz, DMSO-*d*_6_): *δ* = 8.81 (d, 6H, *J* = 8.0 Hz), 8.40 (s, 6H), 7.66–7.81 (m, 12H); ESI-MS (MeOH): *m*/*z* = 298.0692 [M-2Cl]^2+^; elemental analysis calcd. for C_36_H_24_N_6_FeCl_2_·2H_2_O (703.40): C 61.47, H 4.01, N 11.95; found: C 62.34, H 4.57, N 11.17.

**[Fe(phen)_2_(CH_3_CH_2_OH)Cl]Cl (2).** Catalyst **2** was synthesized and purified by a procedure similar to that used for **1**, except that 1.81 g (10.06 mmol) of phen was used. Yield: 74% (1.99 g). ^1^H NMR (400 MHz, DMSO-*d*_6_): *δ* = 8.81 (d, 4H, *J* = 8.0 Hz), 8.41 (s, 4H), 7.74 (dt, 8H *J* = 12.3, 5.0 Hz), 4.35 (t, 1H, *J* = 5.1 Hz), 3.38–3.50 (m, 2H), 1.06 (t, 3H, *J* = 7.0 Hz); ESI-MS (MeOH): *m*/*z* = 451.0422 [M-Cl-CH_3_CH_2_OH]^+^; elemental analysis calcd. for C_26_H_22_N_4_OFeCl_2_ (533.23): C 58.56, H 4.16, N 10.51; found: C 59.28, H 4.45, N 10.61.

**Synthesis of 1,3-dimethyl-2-phenyl-2,3-dihydro-1*H*-benzo-[d]-imidazole (BIH).** NaOH (0.85 g, 21.3 mmol), 2-phenylbenzimidazole (2.9 g, 14.9 mmol) and CH_3_I (7.9 g, 55.7 mmol) were dissolved in 20 mL of methanol with stirring for 20 min. The resulting solution was then transferred into a 50 mL Teflon-sealed autoclave and heated directly to 120 °C in an air-blowing thermostatic oven at a ramping rate of 5 °C min^−1^; the temperature was maintained for 12 h. After cooling to room temperature, a white flake crystal (BI^+^I^−^) was obtained by filtration, and washing with deionized water and methanol. Thereafter, the solid sodium borohydride (567 mg, 15 mmol) was added slowly in batches to a solution containing 2.1 g of BI^+^I^−^ (6 mmol) in methanol (80 mL) with stirring under an atmosphere of nitrogen at 273 K. After the reaction mixture was stirred for 2 h, reaction temperature was kept at 298 K, and processed for 1.5 h. Reaction solvent was then removed by distillation under reduced pressure, and 30 mL of deionized water was added. The resulting mixture was extracted with diethyl ether (3 × 30 mL), and the extractive was washed with the saturated aqueous solution of Na_2_S_2_O_3_ and NaCl, respectively. The organic phase was dried over anhydrous MgSO_4_ overnight. The filtration was concentrated to yield a white solid powder. ^1^H NMR spectrum (400 MHz, CDCl_3_): δ 7.84–7.35 (m, 5H), 6.82–6.33 (m, 4H), 4.93 (s, 1H), 2.61 (s, 6H). ESI-MS (MeOH): *m*/*z* = 223.1215 (M + H)^+^.

### 3.3. Characterization

Single-crystal X-ray diffraction. Single crystals of [Fe(phen)_3_]Cl_2_ suitable for X-ray diffraction analysis were grown by slow evaporation of dichloromethane solutions, which allowed their molecular structures and packing modes in the solid state to be studied. CCDC reference number 1,571,123 contains the supplementary crystallographic data for this paper. These data can be obtained free of charge from The Cambridge Crystallographic Data Centre via www.ccdc.cam.ac.uk/data_request/cif.

Instrumentations. ^1^H NMR spectra were recorded on a Bruker Avance 400 spectrometer with chemical shifts (δ, ppm) relative to tetramethylsilane. The mass spectra were determined on a Finnigan LCQ quadrupole ion trap mass spectrometer and a Shimadzu LCMS-IT-TOF mass spectrometer. Elemental analyses were performed on a Vario EL III. The diffraction data of single crystal were collected on a Rigaku R-AXIS RAPID IP X-ray diffractometer using a graphite monochromator with Mo Kα radiation (*λ* = 0.071073 nm) at 113 K. Electrochemical measurements were carried out on a CHI660C electrochemical potentiostat. Dynamic light scattering measurement was carried out on a DynaPro NanoStar (Wyatt Technology Co., US).

### 3.4. Photocatalytic Experiments

The photocatalytic reduction of CO_2_ to CO was conducted under 1 atm of CO_2_ at 298 K. Typically, 4 mL of DMF/TEOA solution (*v/v*, 7:1) containing [Ru(bpy)_3_]Cl_2_, BIH, and Fe-based catalyst was added to a 18.5 mL cuvette sealed with a rubber septum placed on top of a magnetic stirrer. A stream of CO_2_ was then passed into the reaction system for 30 min. White light-emitting diodes (LEDs) (30 × 3 W, λ ≥ 420 nm) were used as the irradiation light source. The LEDs were positioned 3 cm away from the sample, which was kept under continuous stirring at room temperature. The generated gases were analyzed by a gas chromatography (GC-2014C, 5 Å molecular sieve column (3 m × 2 mm), DINJ 50 °C, DTCD 100 °C, column temperature 50 °C, carrier gas flow 30 mL/min). The amounts of products were determined using the external standard method as the basis for quantitative analysis. ^13^C-labelled CO_2_ experiments were performed following the same procedure and the gas products were analyzed by GC-TCD and GC-MS.

## 4. Conclusions

In summary, a highly efficient, visible-light-driven Fe-based catalytic system was established to reduce CO_2_ to CO. Catalyst **2**, with its easily replaced ligands, displayed a catalytic activity twice as large as that of catalyst **1**, suggesting that the generated empty coordinating sites in the catalytic reduction of CO_2_ play a crucial role. Generally, it is required that coordination-saturated Fe(II) complex catalysts proceed photoinduced reaction to generate active sites before forming Fe-CO_2_ adduct, and the overall results are notable because the photodriven initial periods were similar for catalysts **1** and **2**. However, the results obtained from HRMS measurement indicate an interchange mechanism during CO_2_ photoreduction for complex **1** as catalyst. The interrelationships among the components in the catalytic system, especially the effect of electron donor BIH on the contribution of PS to CO and H_2_ generation, was therefore still further studied in detail. Undoubtedly, the higher selectivity and efficiency of the reported system for the visible-light-driven reduction of CO_2_ to CO offers significant potential for carbon-neutral artificial photosynthesis cycles.

## Figures and Tables

**Table 1:** Controlled experimental results from Fe(phen)_3_Cl_2_ (**1**) for photocatalytic conversion of CO_2_ to CO.

Entry	[cat. 1] μM	[ps] mM	CO μmol	H_2_ μmol	Selectivity of CO	TON_CO_
1	0.03	0.67	4.25	2.00	68.0%	35,417
2	0.15	0.67	11.62	1.29	90.0%	19,367
3	0.15	0.00	0.00	0.06	0	0
4	0.00	0.67	2.23	3.09	41.9%	^a^ 0.83
^b^ 5	0.15	0.67	0.00	1.50	0	0
^c^ 6	0.15	0.67	0.00	0.07	0	0
^d^ 7	0.15	0.67	0.00	0.00	0	0
8	3.00	0.67	19.70	2.11	90.3%	1642
9	30	0.67	61.21	5.57	91.7%	510
10	150	0.67	96.23	5.88	94.2%	160

^a^ TON value only determined from the PS; ^b^ Without CO_2_; ^c^ Under dark conditions; ^d^ Without TEOA. Reaction conditions: LED light irradiation for 2 h, 4 mL CO_2_-saturated DMF/TEOA solution (*v*/*v*, 7:1), 0.022 M BIH.

## Supplementary Materials

Supplementary Materials are available online. Figures S1–S9, Tables S1–S2.
