# Supplementary material for: An Unexpected Iron (II)-Based Homogeneous Catalytic System for Highly Efficient CO2-to-CO Conversion under Visible-Light Irradiation
Source: Molecules. 2019 May 16;24(10):1878. doi: 10.3390/molecules24101878 (PMC6571623; doi:10.3390/molecules24101878)
Supplement: Supplementary file 1 [file molecules-24-01878-s001.pdf]

Supplementary Materials

An Unexpected Iron(II)-Based Homogeneous Catalytic System for Highly Efficient CO_2-to-CO Conversion under Visible-Light Irradiation

Zi-Cheng Fu,[1,] Cheng Mi,[1,] Yan Sun,[1] Zhi Yang,[1] Quan-Qing Xu[1] and Wen-Fu Fu[1,2,]*

[1]*College of Chemistry and Engineering, Yunnan Normal University, Kunming 650092, (P.R. China)*

[2]*Key Laboratory of Photochemical Conversion and Optoelectronic Materials and HKU-CAS Joint Laboratory on New Materials, Technical Institute of Physics and Chemistry, Chinese Academy of Sciences, Beijing 100190, (P.R. China)*

Determination and Calculation of Apparent Quantum Yield. Apparent Quantum Yield (Φ) was calculated from the equation:

Φ = (number of CO molecules × 2)/(number of incident photons) × 100%

The numbers of photons absorbed were determined by an irradiance meter (FZ-A Beijing Normal University Photoelectic Instrument Factory).

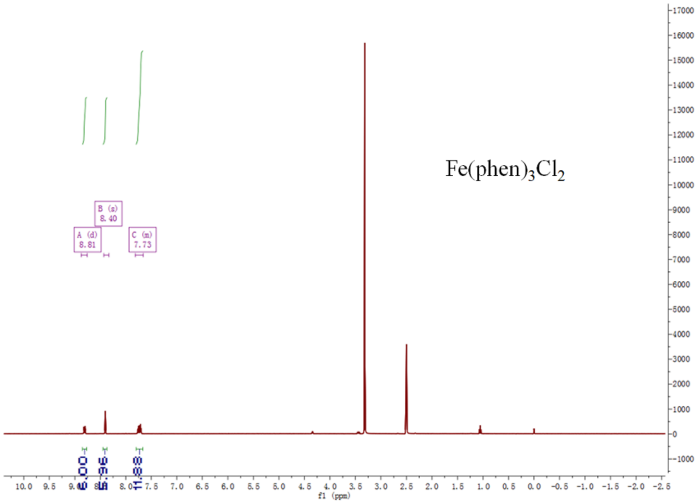
$Fe(phen)_3Cl_2$
A (d) 8.81
B (s) 8.40
C (m) 7.73
f1 (ppm)

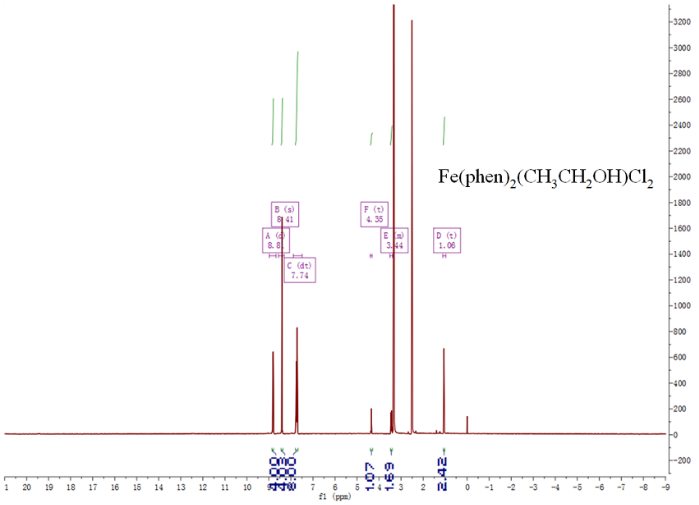
$Fe(phen)_2(CH_3CH_2OH)Cl_2$
B (s) 8.41
C (dt) 7.74
F (t) 4.38
E (m) 3.44
D (t) 1.06
1.07
1.69
2.42
f1 (ppm)

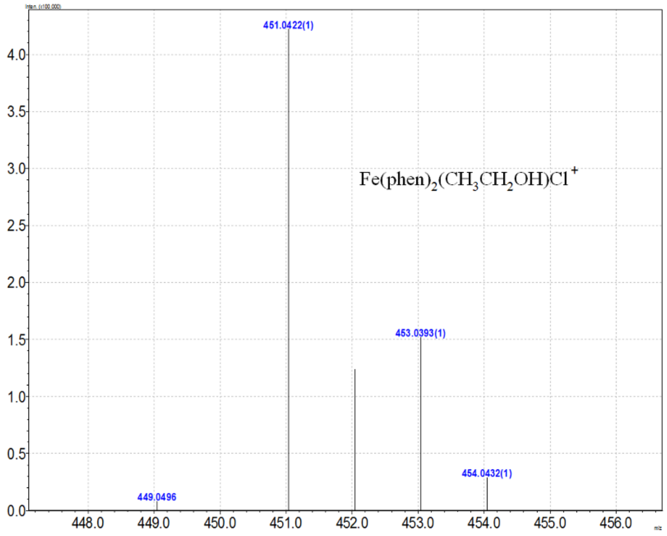
$Fe(phen)_2(CH_3CH_2OH)Cl^+$
451.0422(1)
453.0393(1)
454.0432(1)
449.0496

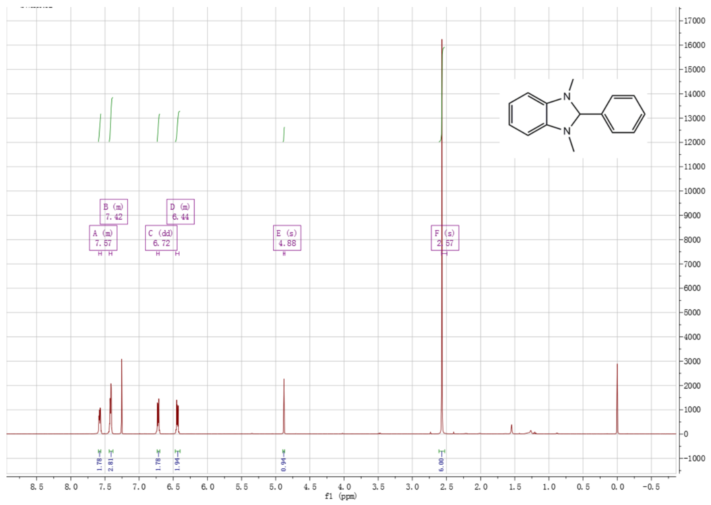

Figure S1. [1]NMR and MS spectra of $Fe(phen)_3Cl_2$ (**1**), $[Fe(phen)_2(CH_3CH_2OH)Cl]Cl$ (**2**) and BIH.

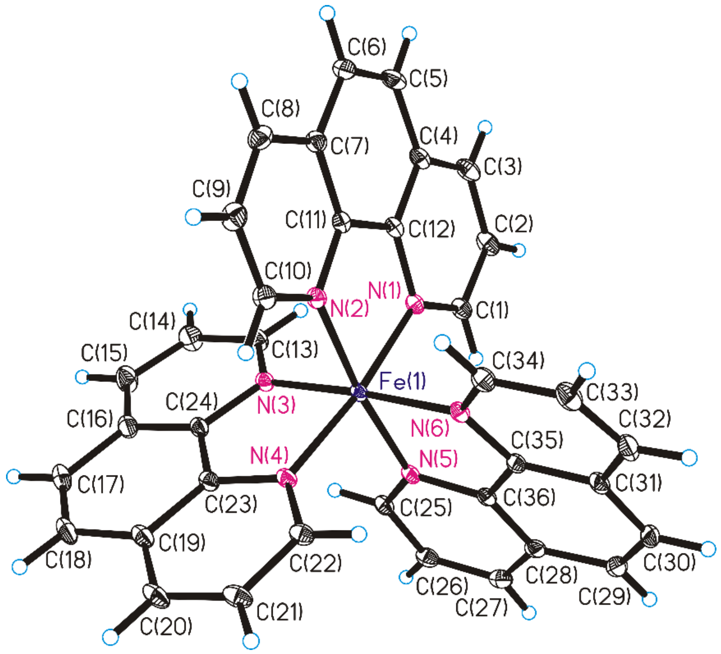

Figure S2. The perspective view and labeling scheme for $Fe(phen)_3^{2+}$ with thermal ellipsoids at 30% probability.

Table S1. X-ray crystallographic date for catalyst **1**.

Compounds	$[Fe(phen)_3]Cl_2$•**2**CH_2Cl_2•**2**H_2O
formula	$C_{38}H_{32}Cl_6FeN_6O_2$
formula weight	873.25
T [K]	113(2)
crystal system	triclinic
space group	P-1
crystal size [mm]	0.20×0.18×0.12
a [Å]	10.786(2)
b [Å]	11.622(2)
c [Å]	16.356(3)
α [°]	96.311(3)
β/ [°]	93.396(2)
γ [°]	113.079(4)
V [Å]^3	1862.9(6)
Z	2
D_c/g cm^{-3}	1.577
μ [mm^{-1}]	0.880
$2\theta_{max}$ [°]	55
unique reflections	8416
parameters	490
R_{int}	0.0397
goodness of fit	1.052
$R1$, w$R2$[I>2$\alpha(I)$]	0.0392, 0.1068
$R1$, w$R2$[all data]	0.0464, 0.1101
max, min peaks (e Å^{-3})	1.063, -1.177

$R1 = \sum||F_o| - |Fc||\sum|F_o|$. $wR_2 = \{\sum[w(F_o^2- F_c^2)^2]/ \sum[w(F_o^2)^2]\}^{1/2}$

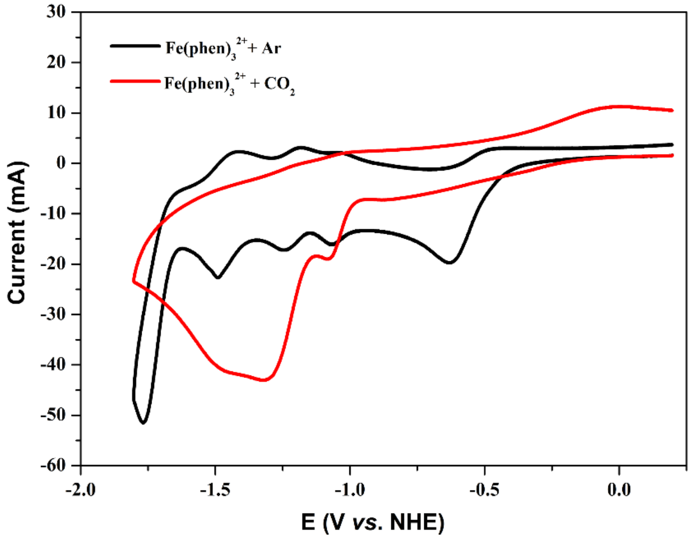

Figure S3. CV of 1 mM $Fe(phen)_3^{2+}$ in DMF solution containing 0.1 M $^nBu_4NPF_6$ under an Ar (black) and CO_2 atmosphere (red) at 25 °C, respectively, using a glassy carbon electrode with a scan rate of 100 mV·s^{-1}.

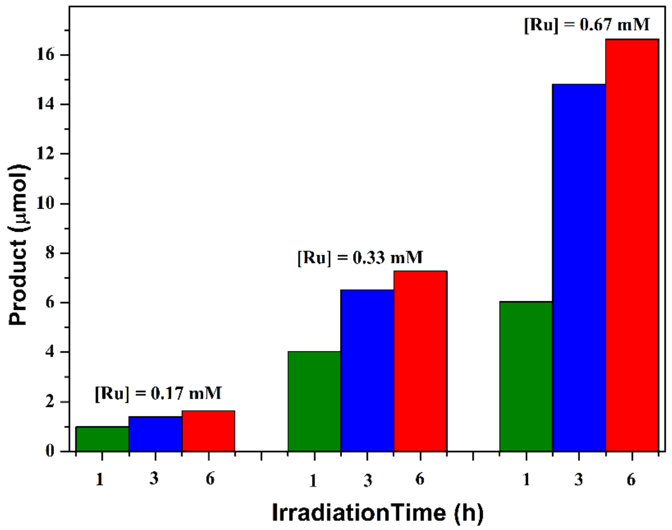

Figure S4. Photosensitizer-concentration and irradiation time dependence of CO production in 4 mL CO_2-saturated DMF/TEOA solution (v/v, 7:1) containing BIH (0.022 M), $Fe(phen)_3Cl_2$ (0.15 μM) and $Ru(bpy)_3^{2+}$ (0.17, 0.33, 0.67 mM) after irradiation for 1, 3 and 6 h at 298 K.

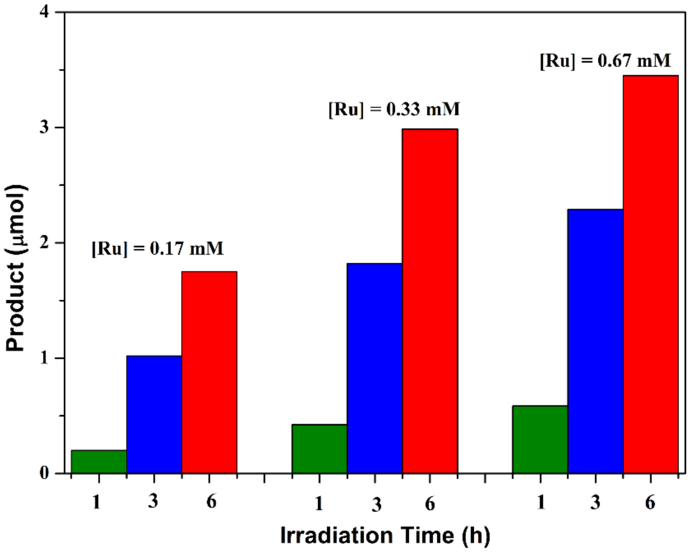

Figure S5. Photosensitizer-concentration and irradiation time dependence of H_2 evolution in 4 mL CO_2-saturated DMF/TEOA solution (v/v, 7:1) containing BIH (0.022 M), $Fe(phen)_3Cl_2$ (0.15 μM) and $Ru(bpy)_3^{2+}$ (0.17, 0.33, 0.67 mM) after irradiation for 1, 3 and 6 h at 298 K.

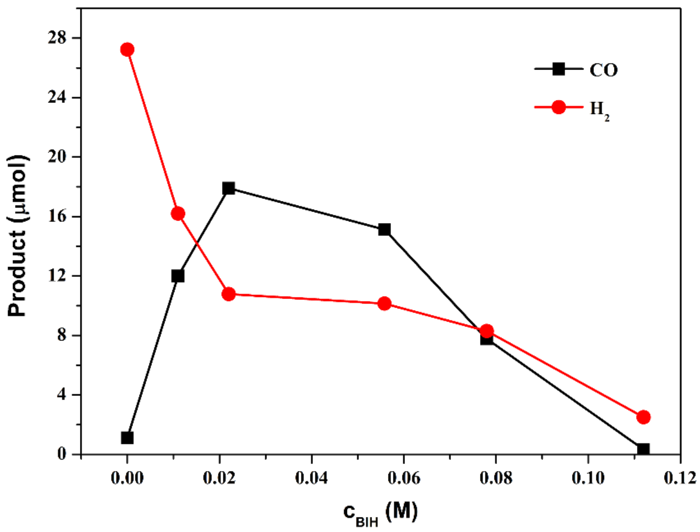

Figure S6 BIH-concentration dependence of CO and H_2 productions in 4 mL CO_2-saturated DMF/TEOA solution (v/v, 2.5:1) containing $Fe(phen)_3Cl_2$ (0.03 μM) and $Ru(bpy)_3^{2+}$ (0.67 mM) after irradiation 13 h at 298 K.

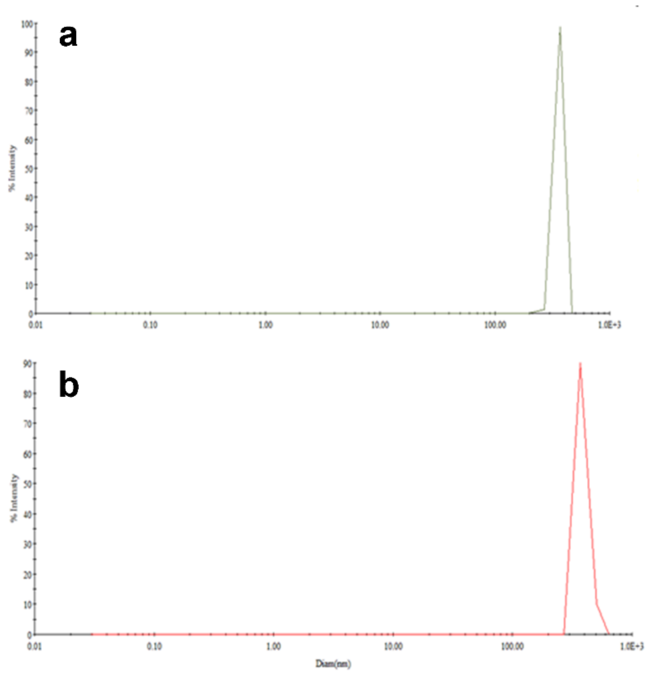

Figure S7. A comparison of DLS results in 4 mL CO_2-saturated DMF/TEOA solution (v/v, 7:1) containing BIH (0.022 M), Fe catalyst (0.15 μM) and $Ru(bpy)_3^{2+}$ (0.67 mM) (a) before and (b) after irradiation for 2 h at 298 K.

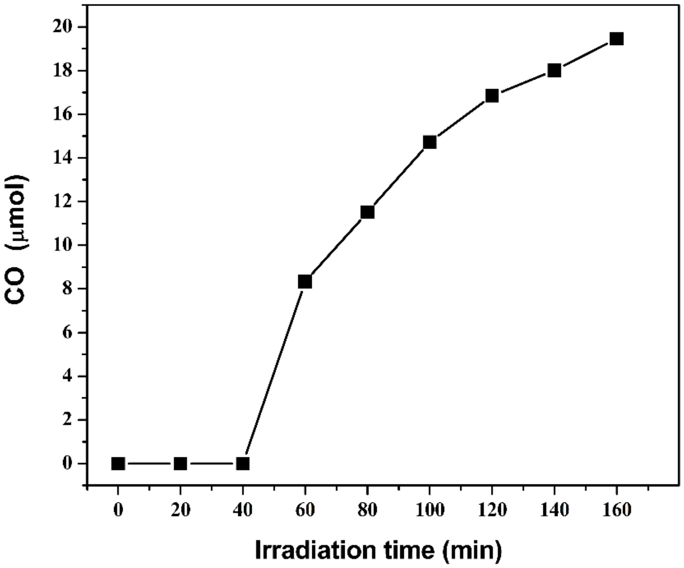

Figure S8. CO evolution of during bright LED visible-light irradiation in 4 mL CO_2-saturated DMF/TEOA solution (v/v, 4:1) containing 6.7 × 10^{-4} M $Ru(bpy)_3^{2+}$, 0.033 M BIH without Fe(II) complex.

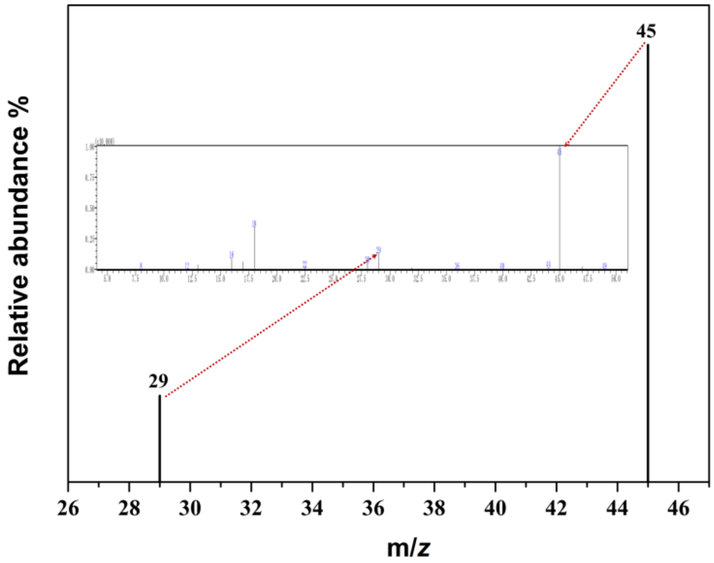

Figure S9. Mass spectra of the gaseous products using $Fe(phen)_3^{2+}$ as catalyst for photocatalytic reduction of $^{13}CO_2$ in the presence of $Ru(bpy)_3^{2+}$ in 4 mL DMF/TEOA solution.

Table S2. Concentration dependence of photocatalytic CO and H_2 evolution on $[Fe(phen)_2(CH_3CH_2OH)Cl]Cl$ (**2**) in 4 mL CO_2-saturated DMF/TEOA solution (v/v, 7:1) containing 6.7×10^{-4} M $Ru(bpy)_3^{2+}$ and 0.022 M BIH after irradiation for 2 h at 298 K.

[cat. 2] μM	[ps] mM	CO μmol	H_2 μmol	Selectivity of CO
30	0.67	119.86	20.56	85.4%
15	0.67	63.93	9.14	87.5%
3.00	0.67	30.80	4.29	87.8%
0.15	0.67	19.90	0.99	95.3%
